# Epidemiology and outcomes of *Candida* spp. bloodstream infections in cancer patients: a comparative retrospective study from a German tertiary cancer center

**DOI:** 10.1007/s15010-025-02513-z

**Published:** 2025-04-02

**Authors:** Sebastian Wolf, Sarah Weber, Aaron Janetta, Friederike Klein, Julius C. Enssle, Michael Hogardt, Volkhard A. J. Kempf, Johanna Kessel, Maria J. G. T. Vehreschild, Björn Steffen, Thomas Oellerich, Hubert Serve, Sebastian Scheich

**Affiliations:** 1 Department of Medicine II-Hematology and Oncology, Goethe-University Frankfurt, University Hospital, Theodor-Stern-Kai 7, 60590 Frankfurt, Germany; 2 Institute for Medical Microbiology and Infection Control, Goethe-University Frankfurt, University Hospital, Theodor-Stern-Kai 7, 60590 Frankfurt, Germany; 3 Department of Medicine II-Infectious Diseases, Goethe-University Frankfurt, University Hospital, Theodor-Stern-Kai 7, 60590 Frankfurt, Germany; 4 Frankfurt Cancer Institute (FCI), Frankfurt, Germany; 5 German Cancer Consortium (DKTK), Partner Site Frankfurt/Mainz, a Partnership Between DKFZ and University Hospital, Frankfurt, Germany; 6 University Cancer Center (UCT), Goethe-University Frankfurt, University Hospital, Theodor-Stern-Kai 7, 60590 Frankfurt, Germany

**Keywords:** *Candida*, Bloodstream infection, Hematology, Oncology, Neutropenic fever, Acute leukemia

## Abstract

**Background:**

Bloodstream infections (BSI) due to *Candida* spp. significantly contribute to morbidity and mortality among cancer patients. Understanding their clinical course, risk factors, and outcomes compared to bacterial BSI is essential.

**Aim:**

We aim to elucidate the epidemiology and risk factors associated with *Candida* BSI compared to bacterial BSI in cancer patients.

**Methods:**

We analyzed epidemiological data of *Candida* BSI versus bacterial BSI among cancer patients, primarily with hematological malignancies. Blood cultures were obtained upon clinical suspicion, with species identification by VITEK 2 and MALDI-TOF. Susceptibility testing utilized VITEK 2 or antibiotic gradient tests.

**Results:**

*Candida* BSI was associated with higher 30-day mortality compared to bacterial BSI (Hazard ratio (HR) 4.5, 95% CI 2.5–8.1, p < 0.001) occurring predominantly in patients with relapsed/refractory disease. Univariate analysis identified risk factors for *Candida* BSI: hypoalbuminemia (Odds ratio (OR) 9.13, 95% CI 2.7–57, p = 0.003), prior ICU/MC stay (OR 3.91, 95% CI 1.38–9.65, p = 0.005), palliative treatment (OR 3.42, 95% CI 1.52–7.4, p = 0.002), parenteral nutrition (OR 2.44, 95% CI 0.9–5.5, p = 0.039) and prior allogeneic HSCT (OR 2.28, 95% CI 0.92–5.13, p = 0.056). Risk factors identified by multivariate analysis were palliative therapy (OR 5.23, 95% CI 3.14–8.71, p = 0.001), hypoalbuminemia (OR 9.02, 95% CI 4.23–19.2, p = 0.004), and prior ICU/IMC stay (OR 4, 95% CI 2.31–6.92, p = 0.011). In patients with confirmed *Candida* BSI, delayed initiation of antifungal was associated with worse outcomes.

**Conclusion:**

Compared to bacterial BSI events, *Candida* BSI are associated with significantly higher 30-day mortality, primarily affecting heavily pretreated patients with relapsed or refractory disease.

**Supplementary Information:**

The online version contains supplementary material available at 10.1007/s15010-025-02513-z.

## Introduction

### Background

Bloodstream infections (BSI) are a major cause of mortality and morbidity in patients with cancer and severe hematological diseases, accounting for 20–30% of all febrile episodes during neutropenia [1, 2]. In bacterial BSI, gram-negative organisms like *Escherichia coli* are more frequent than gram-positive species such as *Entereococcus* spp. and infections with antibiotic-resistant bacteria are associated with increased mortality [3]. Cancer patients, both with solid tumors and hematological malignancies, as well as those with severe non-malignant hematologic diseases like severe aplastic anemia, face particularly high BSI risk due to disease- and treatment-related immunosuppression [4–6]. This risk is further elevated by the necessity of indwelling venous catheters for therapy administration.

Fungal BSI are less frequent, with reported rates of 0.20–0.38 per 1,000 admissions, primarily affecting surgical patients and those with severe immunosuppression, including intensive-care unit (ICU) patients, and cancer patients, particularly in the setting of an allogeneic stem cell transplantation [4, 5]. While *Candida albicans* accounts for approximately 60% of fungal BSI, non-albicans species with reduced azole susceptibility are increasingly prevalent, particularly among hematological patients receiving broad-spectrum azole prophylaxis [6–8]. Due to their low incidence, comprehensive data on the epidemiology, outcomes, and risk factors of *Candida* BSI remain limited, and to date, no studies have directly compared fungal and bacterial BSI in cancer patients.

This study makes several important contributions to the current literature on *Candida* BSI. It confirms the significantly poorer overall survival in this vulnerable patient population and highlights both well-established and previously unrecognized clinical risk factors. These findings provide actionable insights that can be directly integrated into clinical practice to enhance patient management and outcomes.

### Objectives

In this single-center retrospective study at a German tertiary university center, we aimed to elucidate the epidemiology and associated risks of *Candida* BSI compared to bacterial BSI in a large cohort of hematological and oncological patients. Additionally, we sought to identify risk factors for developing *Candida* BSI and evaluate the antimicrobial treatment strategies employed in these patients.

## Methods

### Study design and data collection

This study analyzed 637 BSI events occurring in 403 patients with hematological and oncological diseases between September 2006 and January 2023 at the University Hospital in Frankfurt, Germany. Patients were identified through screening for bacterial and *Candida* BSI reports from hospitalized patients in the Department of Medicine, Hematology, and Oncology. Clinical data were extracted from electronic medical records. The study was approved by the local ethics committee (approval number: SHN-10–2017).

The primary endpoint was defined as overall survival within 30 days after a BSI event (30-day OS). Secondary endpoints included the identification of risk factors associated with *Candida* BSI development, characterization of therapeutic sequences, and comparison of mortality rates between *Candida* and bacterial BSI. Findings on bacterial BSI outcomes in this cohort have been previously reported [9].

### Microbial testing and definitions

Blood cultures were obtained at the treating physicians' decision when patients presented with fever or other signs of systemic infection. Species identification (bacteria and *Candida* spp.) was done by VITEK 2 (bioMérieux, Nürtingen, Germany) and since 2010 by matrix-assisted laser desorption ionization-time of flight analysis (VITEK MS, bioMérieux). Antibiotic susceptibility testing was performed according to Clinical and Laboratory Standards Institute (CLSI) guidelines and since 2019 according to the recommendations of the European Committee on Antimicrobial susceptibility testing (EUCAST) using VITEK 2 and/or antibiotic gradient tests (bioMérieux) for bacteria and VITEK 2 (AST-YS 07/08) and/or Micronaut microdilution assay (Bruker Daltronics GmbH, Germany) for *Candida* spp. [10]. In the case of carbapenem-resistant *Enterobacteriaceae* or *Acinetobacter baumannii*, detection of genes encoding carbapenemases (i.e., NDM, VIM, IMP, OXA-48 like, and KPC for Enterobacteriaceae as well as OXA–23, OXA–24, OXA–58 and NDM for *Acinetobacter baumannii*) was routinely performed via PCR analysis and subsequent sequencing in line with published guidelines [11, 12]. BSI was defined as the detection of at least one bacterial or fungal organism in one blood culture. For common skin commensals (CSC), including coagulase-negative *Staphylococcus* spp., *Bacillus* spp., *Corynebacterium* spp., *Cutibacterium* spp., and *Micrococcus* spp., two positive blood cultures within 48 h were required to fulfill the BSI definition. MDRGN was defined as *Enterobacterales*, *Acinetobacter baumannii*, or *Pseudomonas aeruginosa* showing resistance to at least three out of four antibiotic classes: piperacillin as indicator agent for penicillins, cefotaxime and/or ceftazidime as indicator agent for cephalosporins, imipenem and/or meropenem as indicator agents for carbapenems and ciprofloxacin as indicator agent for fluoroquinolones, as described previously [3, 13, 14]. Organisms resistant to defined agents of all four antibiotic classes, including carbapenems, were classified as MDRGN + CR. The collective term multidrug-resistant organisms (MDRO) encompassed MDRGN, MDRGN + CR, vancomycin-resistant *Enterococci* (VRE), and methicillin-resistant *Staphylococcus aureus* (MRSA). For subsequent analysis, bacterial species were grouped according to their genera and antibiotic resistance profiles. Rare organisms (RO) were categorized based on their bacterial family, obligate anaerobic growth behavior, and gram staining. All microbiological analyses were performed as previously described [3]. In cases where patients experienced both bacterial and *Candida* BSI, only the *Candida* BSI event was analyzed, while additional bacterial BSI events from these patients were excluded.

### Antimicrobial treatment

Antimicrobial prophylaxis with levofloxacin was routinely administered to patients with anticipated prolonged neutropenia (≥ 10 days). Cotrimoxazole/trimethoprim prophylaxis was standard for patients undergoing hematopoietic stem cell transplantation (HSCT), those with acute lymphoblastic leukemia (ALL), lymphomas, or other conditions associated with increased risk for *Pneumocystis jirovecii* pneumonia [15].

HSCT patients received additional prophylaxis comprising levofloxacin or cefotaxime, a broad-spectrum azole (e.g. voriconazole or posaconazole), and acyclovir. During acute myeloid leukemia (AML) induction chemotherapy, patients received prophylaxis with levofloxacin and a broad-spectrum azole, typically posaconazole.

Upon presentation with clinical signs of infection, empiric antibiotic therapy was initiated: neutropenic patients routinely received piperacillin/tazobactam, while patients with known MDRGN colonization received imipenem or meropenem. For patients colonized with MRDGN + CR, empirical antimicrobial therapy was guided by the organism's available resistance patterns. The selection of reserve group antibiotics, including colistin, ceftazidime-avibactam, or cefiderocol, was determined based on the suspected infection site and the severity of clinical presentation. Antibiotic treatment was adjusted as necessary following microorganism identification in patient samples.

### Statistical analysis

Categorical variables were analyzed using the Chi-squared test when all expected cell counts were ≥ 5; otherwise, Fisher's exact test was applied. Continuous variables were compared using a two-tailed Wilcoxon rank-sum test. Results were considered significant at p < 0.05. The 30-day overall survival was estimated using the Kaplan–Meier method, with differences assessed by the log-rank test. Factors potentially impacting 30-day OS were evaluated using univariate Cox regression models Risk factors for *Candida* BSI were identified using logistic regression with *Candida* BSI as a binary response variable. Variables with more than two levels were converted to binary format using one-hot encoding. Variables with predictors showing p < 0.1 were subsequently included in a multivariate model. All analyses were performed using R version 4.3.2 (https://www.r-project.org). Survival analyses were performed using survival (v. 3.5–7) and survminer (v. 0.4.9), plots were created using ggplot2 (v. 3.5.1).

## Results

### Patient characteristics

To characterize the epidemiology of *Candida* BSI in a large cohort of hematological and oncological patients and compare outcomes and risk factors with bacterial BSI, we analyzed 637 individual BSI events in 403 patients. Of these events, 29 (4.5%) were *Candida* BSI, while the remainder were bacterial BSI. Of these patients, 159 (39.5%) were male, with a median age of 53.9 years. The majority had hematological malignancies (n = 369, 91.5%), while the remainder were diagnosed with solid cancers or non-malignant hematological diseases (Table 1).

**Table 1:** Patient demographics and disease characteristics

	Overall	Bacterial BSI	*Candida* BSI	p-value
	n = 403	n = 374	n = 29	
Sex = female (%)	159 (39.5)	145 (38.8)	14 (48.3)	0.329
Age at first diagnosis (mean (SD))	53.89 (14.54)	53.71 (14.80)	56.21 (10.54)	0.452
Disease (%)				0.374
Acute lymphoblastic leukemia	49 (12.2)	43 (11.5)	6 (20.7)	
Acute myeloid leukemia	194 (48.1)	182 (48.7)	12 (41.4)	
Myeloproliferative neoplasia	14 ( 3.5)	14 ( 3.7)	0 ( 0.0)	
Lymphoid neoplasia^a^	102 (25.3)	95 (25.4)	7 (24.1)	
Myelodysplastic syndrome	10 ( 2.5)	9 ( 2.4)	1 ( 3.4)	
Non-malignant condition^b^	14 ( 3.5)	14 ( 3.7)	0 ( 0.0)	
Solid cancer	20 ( 5.0)	17 ( 4.5)	3 (10.3)	

^a^lymhoid neoplasia include Hodgkin lymphoma, high-grade and indolent non-Hodgkin B-cell lymphoma, T-cell lymphoma and plasma cell dyscrasia

^b^non-malignant condition include agranulocytosis, (very) severe aplastic anemia, immune thrombocytopenia, pernicious anemia, sickle cell disease and autoimmune hemolytic anemia

A total of 637 bloodstream infection (BSI) events were analyzed in 403 patients with hematological and oncological diseases. Patient and disease characteristics are presented for the entire cohort (overall column) and stratified by bacterial and *Candida* BSI. Categorical variables are reported as counts and relative frequencies (%), while continuous variables are presented as means with standard deviations (SD). Group comparisons were performed using the two-tailed Wilcoxon rank-sum test for continuous variables and Fisher’s exact test for categorical variables. Reported p-values indicate statistical significance

At the time of BSI events, 289 (45.4%) patients were receiving first-line treatment, while 199 (31.2%) and 149 (23.4%) events occurred during second or later lines of treatment, respectively. BSI events occurred during the first 100 days after allogeneic HSCT in 162 cases (25.4%) and after autologous HSCT in 44 cases (6.9%) (Table 2).

**Table 2:** Comparison of clinical and disease-associated factors between bacterial and *Candida* BSI events

	Overall	Bacterial BSI	*Candida* BSI	p-value
	n = 637	n = 608	n = 29	
Disease factors				
Disease status (%)				**0.005**
First diagnosis	328 (51.5)	321 (52.8)	7 (24.1)	
Remission	58 ( 9.1)	53 ( 8.7)	5 (17.2)	
Relapsed or refractory	251 (39.4)	234 (38.5)	17 (58.6)	
Palliative treatment (%)	103 (16.2)	92 (15.1)	11 (37.9)	**0.003**
Therapy line (%)				**0.015**
First line	289 (45.4)	283 (46.5)	6 (20.7)	
Second line	149 (23.4)	138 (22.7)	11 (37.9)	
Later lines	199 (31.2)	187 (30.8)	12 (41.4)	
Stem cell transplantation within 100 days (%)				0.768
Allogeneic	162 (25.4)	156 (25.7)	6 (20.7)	
Autologous	44 ( 6.9)	43 ( 7.1)	1 ( 3.4)	
No SCT	431 (67.7)	409 (67.3)	22 (75.9)	
Stem cell transplantation beyond 100 days (%)				**0.026**
Allogeneic	95 (14.9)	87 (14.3)	8 (27.6)	
Autologous	8 ( 1.3)	7 ( 1.2)	1 ( 3.4)	
Allogeneic and autologous	33 ( 5.2)	30 ( 4.9)	3 (10.3)	
No SCT	501 (78.6)	484 (79.6)	17 (58.6)	
Clinical factors				
Comorbidities/CCI (%)				
Diabetes mellitus	87 (13.7)	81 (13.3)	6 (20.7)	0.266
HIV	19 ( 3.0)	19 ( 3.1)	0 ( 0.0)	1.000
Liver disease	43 ( 6.8)	41 ( 6.7)	2 ( 6.9)	1.000
Renal failure	66 (10.4)	62 (10.2)	4 (13.8)	0.529
Heart disease	140 (22.0)	128 (21.1)	12 (41.4)	**0.019**
Lung disease	59 ( 9.3)	53 ( 8.7)	6 (20.7)	**0.043**
ICU/IMC admission within 90 days prior to BSI (%)	44 ( 6.9)	38 ( 6.2)	6 (20.7)	**0.011**
ICU/IMC stay during BSI (%)	63 ( 9.9)	58 ( 9.5)	5 (17.2)	0.194
ICU/IMC admission because of sepsis within 30d after BSI (%)	72 (11.3)	65 (10.7)	7 (24.1)	**0.035**
Central venous catheter at the time of BSI (%)	523 (82.1)	498 (81.9)	25 (86.2)	0.804
No corticosteroids at the time of BSI (%)	352 (57.7)	336 (57.8)	16 (55.2)	0.848
Parenteral nutrition at the time of BSI (%)	90 (14.1)	82 (13.5)	8 (27.6)	0.05
Immunosuppressive therapy at the time of BSI (%)	201 (31.6)	192 (31.6)	9 (31.0)	1.000
Chemotherapy within 30 days prior to BSI (%)	497 (78.0)	477 (78.5)	20 (69.0)	0.251
Mucositis grade III/IV at the time of BSI (%)	71 (11.1)	67 (11.0)	4 (13.8)	0.553
Laboratory markers				
C-reactive protein [mg/dl] at the time of BSI (mean (SD))	9.45 (9.24)	9.25 (9.15)	13.61 (10.21)	**0.011**
Creatinine above ULN (%)	125 (19.6)	121 (19.9)	4 (13.8)	0.631
Albumin [g/dl] (mean (SD))	3.27 (0.57)	3.30 (0.56)	2.71 (0.57)	** < 0.001**
Quick [percentage] (mean (SD))	80.11 (18.51)	80.33 (18.62)	75.52 (15.76)	0.159

A total of 637 bloodstream infection (BSI) events were analyzed. Clinical context, patient characteristics, and disease factors at the time of the BSI event are presented for the entire cohort (overall column) and stratified by bacterial and *Candida* BSI. Categorical variables are reported as counts and relative frequencies (%), while continuous variables are presented as means with standard deviations (SD). Group comparisons were performed using the two-tailed Wilcoxon rank-sum test for continuous variables and Fisher’s exact test for categorical variables. Reported p-values indicate statistical significance, p < 0.05 are written in bold. *ULN* upper limit of normal, *SCT* stem cell transplantation, *ICU* intensive-care unit, *IMC* intermediate-care unit, *SD* standard deviation, *BSI* blood stream infection

Among the 608 bacterial BSI events, common skin commensals (CSC) were most frequently isolated (24.8%), followed by *E. coli* (23%), *Enterococci* (22.5%), *Pseudomonas* spp. (5.9%), and *Klebsiella* spp. (5.1%) (Table S1). Of the 29 *Candida* BSI events, *Candida albicans* was detected in 9 patients (31%), *Candida glabrata* in 7 (24.1%), *Candida krusei* in 5 (17.2%), and *Candida dubliniensis* or *Candida tropicalis* in 4 patients each (13.8%). Although not statistically significant, *C. albicans* BSI was less frequent than non-albicans *Candida* BSI in AML patients (n = 2 vs. n = 10), possibly due to azole prophylaxis during induction chemotherapy.

### Clinical impact of *Candida* BSI compared to bacterial BSI

**Fig. 1:**
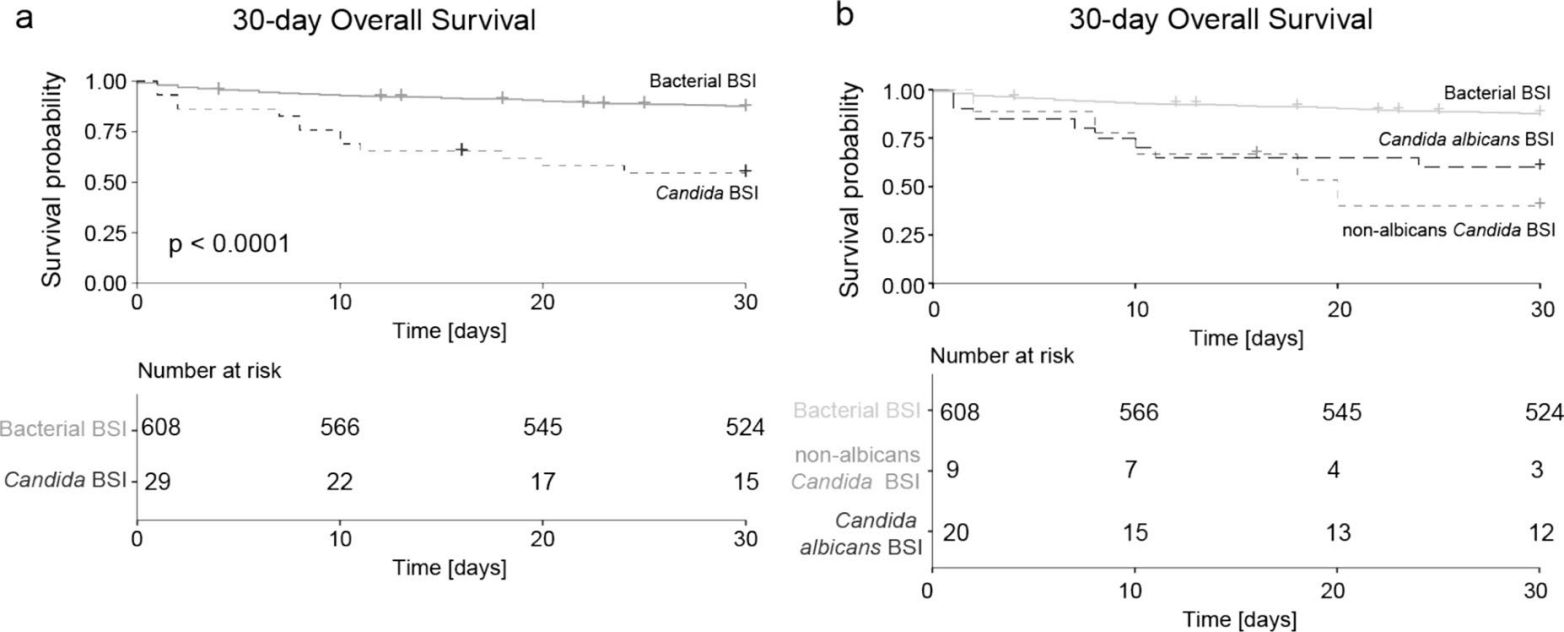
Overall 30-day survival and mortality risk following bloodstream infections** a** Kaplan–Meier curves depicting 30-day overall survival after bloodstream infection, comparing *Candida* infections (dashed line) with bacterial infections (solid line). The corresponding risk table is shown, and the p-value is calculated using the log-rank test. **b** Kaplan–Meier curves for 30-day overall survival, comparing *Candida albicans* (dashed dark grey line), non-albicans *Candida* species (dashed light grey line), and bacterial infections (solid line). The corresponding risk table is included, with p-values determined by the log-rank test. *BSI* blood stream infection

The 30-day OS was significantly worse in patients with *Candida* BSI compared to those with bacterial BSI (log-rank p < 0.0001) (Fig. 1a and Table S2). No difference in 30-day OS was observed between albicans and non-albicans BSI (Fig. 1b). Univariate analysis revealed that *Candida* BSI was associated with a hazard ratio (HR) of 4.48 (95% CI 2.48–8.07, p < 0.001) compared to bacterial BSI.

Patients with *Candida* BSI more frequently required intensive or intermediate care unit admission for sepsis treatment during the 30 days following BSI (24.1%, n = 7 vs. 10.7%, n = 65 for bacterial BSI; p = 0.035, Table 2). Laboratory diagnostics showed significantly higher C-reactive protein (CRP) levels in the *Candida* BSI cohort (mean 13.61 mg/dL vs. 9.25 mg/dL in bacterial BSI; p = 0.011) at the time of BSI.

**Fig. 2:**
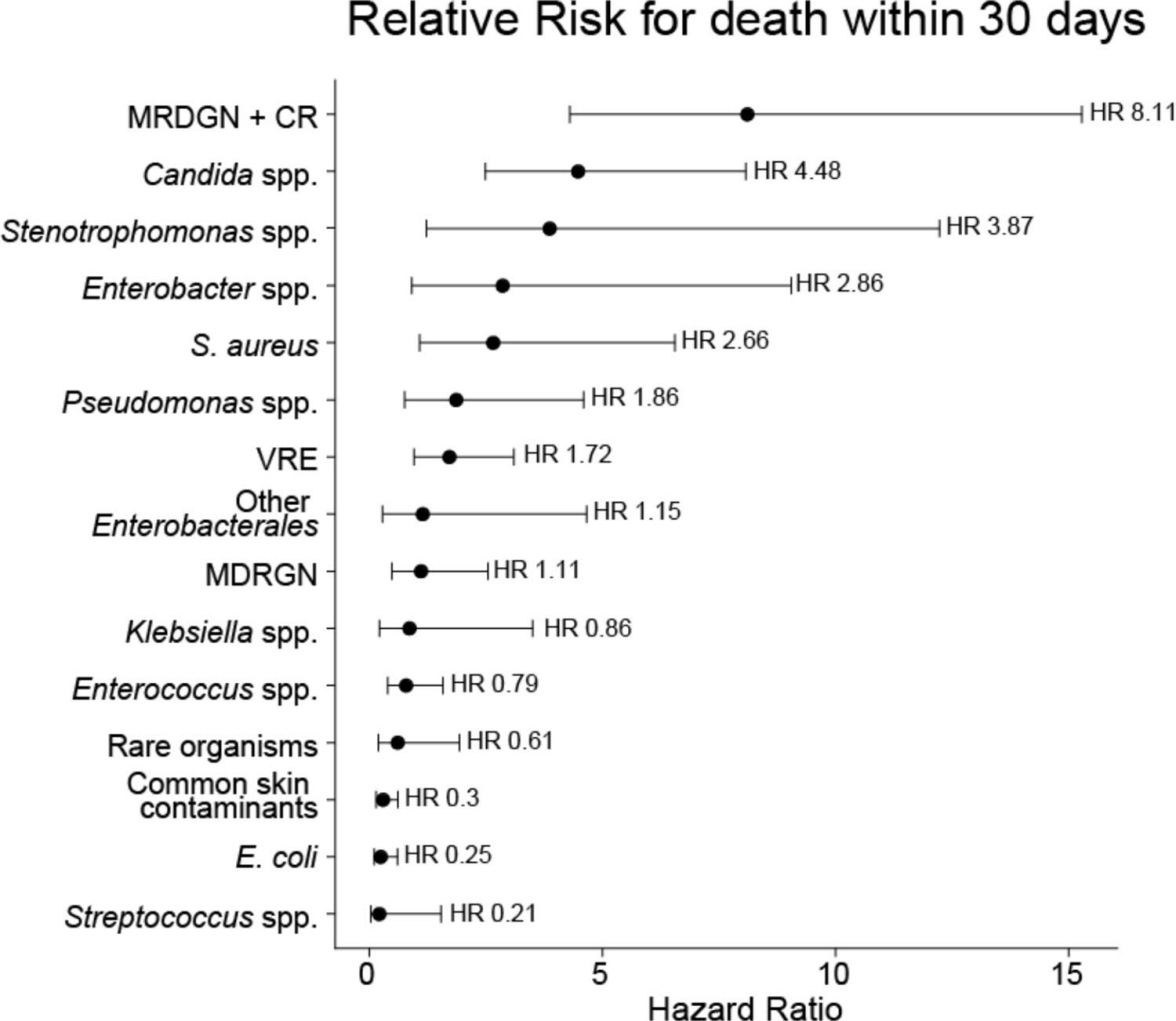
Forest plot of mortality risk within 30 days after BSI, comparing different bacterial and *Candida* isolates. Hazard ratios were calculated using univariate Cox proportional hazard models and ranked in descending order. Points represent HR estimates, with error bars indicating 95% confidence intervals (2.5th to 97.5th percentiles). *VRE* vancomycin-resistant *Enterococci*, *MRDGN* multidrug-resistant gram-negative organisms, *CR* carbapenem resistance

To directly compare the risk associated with different BSI organisms, including antimicrobial drug resistance profiles, we ranked BSI organisms by their estimated 30-day OS HR using Cox regression models (Fig. 2 and Table S3). We compared MDRGN with carbapenem resistance (MDRGN + CR group), VRE, and MDRGN without carbapenem resistance (MDRGN group). Notably, MDRGN + CR BSI was associated with the highest mortality within 30 days after the BSI event (HR 8.11, 95% CI 4.3–15.3, p < 0.001), followed by *Candida* BSI (HR 4.48, 95% CI 2.5–8.1, p < 0.001).

### Risk factors for the development of *Candida* BSI

Compared to bacterial BSI, patients with *Candida* BSI were more frequently treated in later therapy lines (79.3%, n = 23 vs. 53.5%, n = 325; p = 0.01, Table 2) and for relapsed or refractory disease (58.6%, n = 17 vs. 38.5%, n = 234; p = 0.048) with palliative intent (37.9%, n = 11 vs. 15.1%, n = 92; p = 0.003). Patients with *Candida* BSI were also more frequently treated in intensive or intermediate care units during the 90 days prior to the BSI event (20.7%, n = 6 vs. 6.2%, n = 38; p = 0.011), and all *Candida* BSI events were classified as nosocomial based on their occurrence later than 48 h upon admission. A trend toward higher frequency of parenteral nutrition was observed in the *Candida* BSI cohort (27.6%, n = 8 vs. 13.5%, n = 82; p = 0.05). Multivariate analysis confirmed *Candida* BSI as an independent predictor of 30-day OS after adjusting for relapsed/refractory disease and palliative treatment (HR 2.37, 95% CI 1.27–4.42, p = 0.006).

Regarding underlying risk factors, the *Candida* BSI cohort showed higher frequencies of cardiac and pulmonary comorbidities as classified by the Charlson comorbidity index (CCI) [16]. Laboratory markers revealed markedly lower blood albumin levels in *Candida* BSI patients (mean 2.71 g/dL vs. 3.30 g/dL, p = 0.011).

Independent logistic regression models with *Candida* BSI as the response variable (Table 3) identified several significant risk factors associated with *Candida* BSI: hypoalbuminemia (OR 9.13, 95% CI 2.7–57.0, p = 0.003), prior ICU or IMC stay (OR 3.91, 95% CI 1.38–9.65, p = 0.005), palliative treatment (OR 3.43, 95% CI 1.52–7.4, p = 0.002), parenteral nutrition (OR 2.44, 95% CI 0.99–5.50, p = 0.039), prior allogeneic HSCT (OR 2.28, 95% CI 0.92–5.13, p = 0.056), relapsed or refractory disease (OR 2.26, 95% CI 1.07–4.94, p = 0.034), and later line treatment (OR 2.08, 95% CI 0.93–4.45, p = 0.063). Factors associated with a lower risk for *Candida* compared to bacterial BSI included no prior stem cell transplantation (OR 0.36, 95% CI 0.17–0.80, p = 0.009), first-line therapy (OR 0.30, 95% CI 0.11–0.70, p = 0.01), and de novo disease (OR 0.28, 95% CI 0.11–0.64, p = 0.004).

**Table 3:** Uni- and multivariate logistic regression for risk of *Candida* BSI

	Coefficient	CI lower	CI upper	p-value	OR	OR CI lower	OR CI upper
Univariate models							
Hypoalbuminemia	2.21	0.99	4.04	0.003	9.13	2.70	57.00
ICU/IMC 90 days prior	1.36	0.32	2.27	0.005	3.91	1.38	9.65
Palliative treatment	1.23	0.42	2.00	0.002	3.43	1.52	7.40
Parental nutrition	0.89	−0.01	1.71	0.039	2.44	0.99	5.50
Allogeneic stem cell transplantation beyond 100 days	0.82	−0.08	1.63	0.056	2.28	0.92	5.13
Relapsed or refractory disease	0.82	0.07	1.60	0.034	2.26	1.07	4.94
Later line therapy	0.73	−0.07	1.49	0.063	2.08	0.93	4.45
No prior stem cell transplantation	−1.01	−1.77	−0.23	0.009	0.36	0.17	0.80
Fist line therapy	−1.21	−2.21	−0.35	0.01	0.30	0.11	0.70
First diagnosis	−1.26	−2.20	−0.44	0.004	0.28	0.11	0.64
Multivariate models							
Hypoalbuminemia	2.20	0.93	4.05	0.004	9.02	2.54	57.62
Palliative treatment	1.65	0.65	2.66	0.001	5.23	1.91	14.33
ICU/IMC 90 days prior	1.39	0.24	2.43	0.011	4.00	1.28	11.32

Risk factors for *Candida* BSI were analyzed using logistic regression, with *Candida* BSI as the binary outcome variable. Categorical variables with more than two levels were transformed into binary indicators using one-hot encoding. The table presents variables in the left-most column, followed by estimated coefficients, their 95% confidence intervals, and p-values. Odds ratios (OR) were derived from the coefficients and are reported in the corresponding column. For multivariate models, variables with p-values < 0.1 were included in the initial selection, and only those with p-values < 0.05 are shown in the table. *ICU* intensive-care unit, *IMC* intermediate-care unit

Multivariate logistic regression analysis of significant factors identified palliative therapy (OR 5.23, 95% CI 1.91 – 14.33, p = 0.001), hypoalbuminemia (OR 9.02, 95% CI 2.54–19.2, p = 0.004), and prior ICU or IMC stay (OR 4, 95% CI 2.31–6.92, p = 0.011) as independent risk factors for *Candida* BSI (Table 3).

### Antimicrobial therapy sequences in *Candida* BSI

The sequence of antimicrobial therapy in *Candida* BSI was evaluated from the time of initial clinical triggers, typically fever or significant elevation in inflammatory markers such as CRP. At BSI onset, the majority of patients (58.6%, n = 17) were receiving antibiotic prophylaxis, and 8 patients (27.6%) were on antimycotic prophylaxis (fluconazole n = 1, posaconazole n = 4, voriconazole n = 2, caspofungin n = 1).

**Fig. 3:**
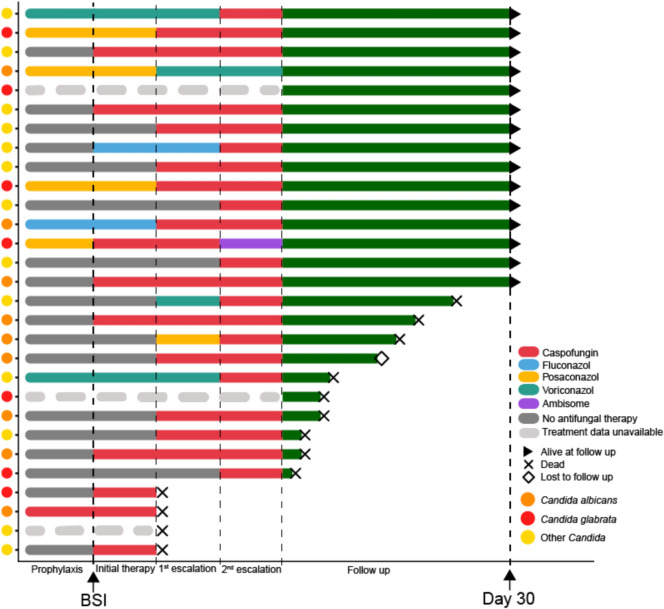
Treatment sequences in patients with *Candida* bloodstream infections. Swimmer plot to displays individual patient treatment trajectories, including antifungal prophylaxis before bloodstream infection (denoted as BSI), initial therapy (first 48 h), first escalation (48–72 h), and subsequent treatment modifications. Green bars indicate the follow-up period (up to 30 days post-BSI), while gray bars represent periods without antifungal therapy. Different colors denote specific antifungal drugs as detailed in the legend. Dots on the y-axis indicate *Candida* species for each patient. Arrowheads show patient outcomes: early death (before 30 days), loss to follow-up, or survival beyond 30 days. Note: Prophylaxis and treatment bar lengths are approximate and do not correspond to daily timescales, while green follow-up bar lengths accurately reflect actual follow-up duration. For patients who died during a therapeutic period, death is indicated at the end of that period. Detailed therapy data is unavailable for three patients as indicated in light grey

The antifungal therapeutic sequence for all 29 *Candida* BSI events is summarized in Fig. 3, detailed data is unavailable for three patients. At BSI onset, empiric antifungal treatment was initiated or modified in 9 patients (31%), while 7 patients continued their established antimycotic prophylaxis. Prior to definitive microbial typing, an additional 11 patients (37.9%) received or were switched to empirical antimycotic therapy during the first treatment escalation, typically prompted by clinical deterioration, lack of improvement, or preliminary blood culture results. In total, 21 patients (75.9%) received empiric broad-spectrum antimycotic treatment within 48 h of clinical infection onset.

Caspofungin, a broad-spectrum echinocandin, was the empiric antimycotic agent of choice in all cases except one, where fluconazole was initiated. Following confirmation of *Candida* BSI, all patients received caspofungin therapy, expect for one patient that was treated with posaconazole and voriconazole. In two cases, microbial typing and resistance testing identified caspofungin-resistant *C. glabrata*; one patient was switched to liposomal Amphotericin B, while the other succumbed to fungal sepsis.

Although the small sample size precluded statistical significance, fewer deaths within 30 days after *Candida* BSI were observed among patients who received antifungal prophylaxis (2 vs. 6 patients) or early antifungal therapy within 48 h after clinical infection (6 vs. 10 patients).

## Discussion

BSI are a major cause of morbidity and mortality in hematological and oncological patients [6]. While fungal BSI account for only a small fraction of cases, primarily affecting immunocompromised and severely ill patients, data on their epidemiology, risk factors, and outcomes remain limited. In this study, we identified, characterized, and compared a large cohort of hematological and oncological patients with bloodstream infections. Among these patients, mainly treated for hematological malignancies, those with *Candida* BSI demonstrated inferior 30-day survival compared to bacterial BSI (HR 4.48, p < 0.001). This finding confirms and expands upon previous studies that report high 30 and 100 day mortality in patients with *Candida* BSI and a strong association with the underlying disease’s severity [4, 5]. Additionally, while prior studies have focused either on the incidence or the poorer prognosis of fungal BSI, our study is the first to provide a direct and detailed comparison of the clinical outcomes of *Candida* versus bacterial BSI [4]. Furthermore, we identified both well-established and previously unrecognized clinical risk factors, emphasizing the novelty of our findings. These insights can be directly integrated into clinical practice, offering guidance for the management of patients at risk for fungal bloodstream infections.

Notably, within the bacterial BSI cohort, mortality risk varied significantly across bacterial taxa and correlated strongly with antibiotic resistance profiles. MDRGN showed the most adverse outcomes, as has been shown by us and others (HR 8.11, p < 0.001) [3, 9, 17, 18]. Interestingly, *Candida* BSI mortality ranked second, underscoring its dismal prognosis (HR 4.48, p < 0.001).

Patients who developed *Candida* BSI were typically treated in later therapy lines for relapsed or refractory disease with palliative intent, suggesting high underlying susceptibility to systemic infections. While neutropenia rates were comparable between *Candida* and bacterial BSI cohorts, prior therapies, advanced disease, and significantly lower serum albumin levels indicated poorer functional status, malnutrition, frailty, and immune dysfunction as underlying risk factors for *Candida* BSI. These factors likely contributed to the poor outcomes. Conversely, our risk models identified first-line therapy and absence of prior allogeneic stem cell transplantation as protective factors against *Candida* BSI. These risk factors can be directly integrated into clinical practice to assess the risk of fungal infections in cancer patients. They may guide decisions on antifungal prophylaxis before cytotoxic chemotherapy or inform empirical antifungal treatment strategies in cases of infectious complications.

Specifically, in febrile patients with poor performance and nutritional status, our data support the early addition of a broad-spectrum antifungal agent to the empirical anti-infective regimen. In contrast, for otherwise stable febrile cancer patients in earlier treatment lines with good performance status—provided there are no additional risk factors for fungal bloodstream infections such as recent abdominal surgery or prior ICU/IMC stay—a more selective approach may be appropriate. In such cases, delaying antifungal therapy until a fungal infection is confirmed or fever persists beyond 72 h despite broad-spectrum antibiotic coverage may be a viable strategy, as supported by existing literature [19].

A strong trend toward higher rates of parenteral nutrition use was observed in *Candida* BSI patients (p = 0.05), potentially reflecting both poor nutritional status and an independent, modifiable risk factor, as long-term central line parenteral nutrition has been identified as a frequent source of *Candida* BSI [20]. This emphasizes the critical importance of adhering to strict hygiene protocols and central line care guidelines in this high-risk patient population. Similarly, prior ICU or IMC stay emerged as one of the strongest independent risk factors, further supporting the association between *Candida* BSI and severe illness with poor functional status and underlying immune dysfunction (OR 3.91, p = 0.005). These findings highlight the need for heightened vigilance in this patient group, particularly in the context of infectious complications.

A number of studies have described risk factors for the development of and mortality associated with *Candida* BSI. Many of these studies focus on medical or surgical IMC/ICU patients, which biases towards a more severely ill population [21, 22] or analyze candidemia outcomes in unselected medical patients [23, 24]. Direct comparison of risk factors between these studies and our analysis is limited, as factors such as neutropenia, prior or concurrent chemotherapy, or immunosuppressive therapy are much more frequent in our hematological/oncological population than in general medical populations.

Nevertheless, several risk factors appear to be independent of the underlying disease: higher age, higher burden of comorbidities, and delay of antifungal therapy [25, 26]. In critically ill patients, greater disease severity and multiorgan dysfunction were identified as main determinants of *Candid*a BSI mortality [22]. Additionally, indwelling central venous catheters and parenteral nutrition were recurrently identified as risk factors for *Candida* BSI development [27].

Our study confirmed several of these established risk factors: higher burden of comorbidities and greater disease severity (indicated by later treatment lines (OR 2.08, p = 0.063) and palliative treatment (OR 3.41, p = 0.002)), as well as and the need for parenteral nutrition (OR 2.44, p = 0.039). However, common risk factors such as neutropenia, chemotherapy, and immunosuppressive therapy were not identified by our models, likely because these factors were prevalent throughout our study population and similarly increase risk for both *Candida* and bacterial BSI. Interestingly, while we did not include prior antibiotic exposure in our dataset, multiple studies report it as an independent risk factor for *Candida* BSI development. Given the strength of this evidence, antibiotic exposure should be considered when evaluating patients' risks. The substantial impact of prior antibiotic exposure and host susceptibility factors, such as inability to maintain oral intake and prior chemotherapies, likely reflects disturbance of the intestinal barrier, which comprises the intestinal microbiome, epithelial cells, and mucosal immunity. This interpretation is supported by compelling clinical and translational evidence identifying intestinal translocation of *Candida* spp. as the primary source of invasive infections, facilitated by bacterial and fungal dysbiosis and compromised intestinal barrier function [28, 29]. Interventions to assess and protect intestinal barrier integrity in hematological and oncological patients are under active investigation, potentially offering new strategies to reduce the risk of invasive fungal infections [30, 31].

While 25% of patients had established antimycotic prophylaxis at BSI onset and another third received empiric antimycotic therapy, resistance to prophylactic agents was confirmed in only one patient (fluconazole). In three cases, detected *Candida* spp. remained susceptible to the prophylactic agent, suggesting potential issues with oral bioavailability, pharmacokinetics (particularly for posaconazole), or drug-drug interactions. These challenges could be addressed through patient education regarding proper drug administration (e.g., posaconazole with food, voriconazole on an empty stomach) and routine therapeutic drug monitoring (TDM) to ensure early achievement and maintenance of target drug concentrations. [32].

Empiric antimycotic therapy was initiated in one-third of patients alongside antibiotics upon clinical infection signs. Including established prophylaxis, approximately half of the patients had adequate antimycotic coverage at diagnosis. Within 48 h and before final microbial identification, roughly 80% received antimycotic therapy. Among 30-day survivors (n = 15), 10 patients (66.7%) had received either antifungal prophylaxis or empiric antifungal treatment at infection onset. In contrast, of those who died within 30 days (n = 14), only 2 patients (14.3%) had early antifungal coverage, while 6 patients received neither prophylaxis nor antifungal treatment at infection onset. While timely anti-infective therapy is crucial for reducing sepsis mortality [33], these apparent differences in survival based on early antifungal coverage did not reach statistical significance due to the small sample size. The observed poor 30-day overall survival in *Candida* BSI might partly be attributable to delayed initiation of adequate antimycotic therapy in those patients who did not receive immediate empiric coverage or had prophylaxis at BSI onset.

This study, while representing a detailed comparative analysis of *Candida* BSI in hematological and oncological, has several limitations. The nonetheless relatively small number of cases limits statistical power for detecting nuanced differences and risk factors in *Candida* BSI cases, as well as confounders. Additionally, the retrospective, single-center design affects the generalizability of our findings, warranting validation in independent cohorts, ideally through large, multi-center trials. Furthermore, patients often presented with multiple concurrent medical conditions, including underlying disease and treatment-related comorbidities, that complicated determination of definitive causes of death. We addressed this by restricting outcome analysis to 30 days post-infection.

In conclusion, our analysis revealed that *Candida* BSI was associated with significantly poorer 30-day survival compared to bacterial BSI, exceeded only by MDRGN + CR BSI. Patients with *Candida* BSI typically presented with more advanced disease, more frequent palliative treatment, and indicators of poor functional status and malnutrition, notably lower serum albumin. We identified several risk factors: later-line therapy, prior stem cell transplantation, prior IMC/ICU stay, hypoalbuminemia, and parenteral nutrition. Only one-third of patients received empiric antimycotic therapy initially. We strongly recommend maintaining high vigilance for *Candida* BSI, particularly in patients with identified risk factors, and considering early antimycotic therapy even in patients receiving oral prophylaxis.

## Supplementary Information

Below is the link to the electronic supplementary material.

Supplementary file1 (DOCX 18 KB)

## Data Availability

No datasets were generated or analysed during the current study.

## Abbreviations

ALL: Acute lymphoblastic leukemia
AML: Acute myeloid leukemia
BSI: Bloodstream infection
CCI: Charlson comorbidity index
CR: Carbapenem resistance
CRP: C-reactive protein
CSC: Common skin commensals
HR: Hazard ratio
HSCT: Hematopoietic stem cell transplantation
ICU: Intensive-care unit
IMC: Intermediate-care unit
MDGRN: Multidrug-resistant gram-negative organisms
MDRO: Multidrug-resistant organism
MDS: Myelodysplastic syndrome/neoplasm
MPN: Myeloproliferative neoplasm
MRSA: Methicillin-resistant *Staphylococcus aureus*
NHL: Non-Hodgkin lymphoma
OS: Overall survival
RO: Rare organisms
SD: Standard deviation
TDM: Therapeutic drug monitoring
VRE: Vancomycin-resistant *Enterococci*
