# Supplementary material for: Epidemiology and outcomes of Candida spp. bloodstream infections in cancer patients: a comparative retrospective study from a German tertiary cancer center
Source: Infection. 2025 Apr 2;53(5):1875–85. doi: 10.1007/s15010-025-02513-z (PMC12460568; doi:10.1007/s15010-025-02513-z)
Supplement: Supplementary file 1 — Supplementary file1 (DOCX 18 KB) [file 15010_2025_2513_MOESM1_ESM.docx]

**Table S1** Microbial isolates in bacterial and fungal BSI Microbial isolates from 637 bloodstream infections (BSI), presented with absolute counts (n) and relative frequencies (%)

| Microbial Isolate | n | (%) |
| --- | --- | --- |
| **Gram-negative bacteria** | **377** | **59.2** |
| E. coli | 140 | 22.0 |
| Enterococcus spp. | 137 | 21.5 |
| Pseudomonas spp. | 36 | 5.7 |
| Klebsiella spp. | 31 | 4.9 |
| Other Enterobacterales | 14 | 2.2 |
| Enterobacter spp. | 11 | 1.7 |
| Stenotrophomonas maltophilia | 8 | 1.3 |
|  |  |  |
| **Gram-positive bacteria** | **197** | **30.9** |
| Common skin commensals (CSC) | 151 | 23.7 |
| Streptococcus spp. | 30 | 4.7 |
| S. aureus | 16 | 2.5 |
|  |  |  |
| **Rare organisms** | **34** | **5.3** |
|  |  |  |
| **Candida albicans** | **9** | **1.4** |
| **non-albicans Candida** | **20** | **3.1** |
| Candida glabrata | 7 | 1.1 |
| Candida krusei | 5 | 0.8 |
| Candida dubliensis | 4 | 0.6 |
| Candida tropicalis | 4 | 0.6 |

**Table S2** Summary of survival analysis at 30 days including total number of patients, number of events (deaths), cumulative survival and mortality with corresponding 95% confidence intervals (CI), and log-rank test p-value comparing groups

|  | Bacterial BSI | *Candida* BSI | p-value |
| --- | --- | --- | --- |
|  | **n=608** | **n=29** |  |
| Events (n) | 76 | 13 |  |
| 30-day survival (%, 95% CI) | 87,4 (84,5-90,1) | 54,6 (39,1-76,3) | <0.0001 |
| 30-day mortality (%, 95% CI) | 12,6 (9,9-15,5) | 45,4 (23,7-60,9) |  |

**Table S3** Impact of microbial isolates on 30-day OS after BSI. Hazard ratios (HR) were estimated using univariate Cox proportional hazard models and are presented in descending order. The table includes 95% confidence intervals (CI) for HR estimates and corresponding p-values. VRE = vancomycin-resistant *Enterococci*, MRDGN = multidrug-resistant gram-negative organisms, CR = carbapenem resistance

| Microbial isolate | HR | HR CI lower | HR CI upper | p-value |
| --- | --- | --- | --- | --- |
| MRDGN + CR | 8.11 | 4.30 | 15.28 | <0.001 |
| Candida spp. | 4.48 | 2.48 | 8.07 | <0.001 |
| Stenotrophomonas spp. | 3.87 | 1.22 | 12.23 | 0.021 |
| Enterobacter spp. | 2.86 | 0.90 | 9.05 | 0.074 |
| S. aureus | 2.66 | 1.08 | 6.55 | 0.034 |
| Pseudomonas spp. | 1.86 | 0.76 | 4.60 | 0.176 |
| VRE | 1.72 | 0.95 | 3.10 | 0.071 |
| Other Enterobacterales^a^ | 1.15 | 0.28 | 4.66 | 0.849 |
| MDRGN | 1.11 | 0.48 | 2.54 | 0.805 |
| Klebsiella spp. | 0.86 | 0.21 | 3.50 | 0.836 |
| Enterococcus spp. | 0.79 | 0.40 | 1.57 | 0.502 |
| Rare organisms^b^ | 0.61 | 0.19 | 1.93 | 0.399 |
| Common skin commensals^e^ | 0.30 | 0.14 | 0.61 | 0.001 |
| E. coli | 0.25 | 0.10 | 0.61 | 0.002 |
| Streptococcus spp. | 0.21 | 0.03 | 1.54 | 0.126 |

**^a^***Serratia marcescens, Morganella morganii, Proteus mirabilis, Citrobacter koseri, Pantoea agglomerans, Raoultella ornithinolytica,* **^b^**rare organisms including *Listeria monocytogenes, Haematobacter massiliensis, Lactobacillus curvatus, Veillonella parvula, Achromobacter xylosoxidans, Rothia mucilaginosa, Bacteroides species, Sphingomonas paucimobilis, Veillonella atypica, Lactobacillus rhamnosus, Ruminococcus gnavus, Capnocytophaga sputigena, Lactococcus lactis, Okibacterium fritillariae, Schaalia odontolyticus, Haemophilus influenzae, Sphingobacterium thalpophilum, Actinomyces neuii, Dermabacter hominis, Leptotrichia buccalis, Bacteroides caccae, Neisseria flava, Granulicatellaadiacens,* **^e^**coagulase-negative *Staphylococci* and *Corynebacterium jeikeium*
